# Molecular Function of cGAS-STING in SARS-CoV-2: A Novel Approach to COVID-19 Treatment

**DOI:** 10.1155/2022/6189254

**Published:** 2022-11-22

**Authors:** Maryam Moossavi, Mandana Rastegar, Seyedeh Zahra Moossavi, Milad Khorasani

**Affiliations:** ^1^Department of Biochemistry and Molecular Biology, Mayo Clinic, Rochester, MN, USA; ^2^Department of Cardiovascular Medicine, Mayo Clinic, Rochester, MN, USA; ^3^Department of Molecular Medicine, Birjand University of Medical Sciences, Birjand, Iran; ^4^Faculty of Medicine, Shiraz University of Medical Sciences, Shiraz, Iran; ^5^Department of Basic Medical Sciences, Neyshabur University of Medical Sciences, Neyshabur, Iran

## Abstract

Coronavirus illness 2019 is a significant worldwide health danger that began with severe acute respiratory syndrome coronavirus two infections. It is the largest pandemic of our lifetime to date, affecting millions of people and crippling economies globally. There is currently no viable therapy for this devastating condition. The fast spread of SARS-CoV-2 underlines the critical need for favorable treatments to prevent SARS-CoV-2 infection and dissemination. Regulating the upstream cytokine release might be a possible method for COVID-19 therapy. We propose that more consideration be paid to the dysregulated IFN-I release in COVID-19 and that cGAS and STING be considered therapeutic targets for avoiding cytokine storms and as critical components in host antiviral defense mechanisms.

## 1. Introduction

Coronaviruses are a kind of RNA virus that causes severe and prolonged upper and lower respiratory tract illnesses in human beings and further animals. Severe acute respiratory syndrome coronavirus 2 (SARS-CoV-2) is a newly arisen coronavirus that began spreading in Wuhan, Hubei Province, China in the last month of 2019 and, as a highly infectious and pathogenic virus, has resulted in a worldwide pandemic, which the World Health Organization (WHO) declared on 11 March 2020 [1]. It is the cause of coronavirus illness 2019, which is a severe respiratory disease (COVID-19) [2].

The disease's spread resembles that of the SARS coronavirus (SARS-CoV) outbreak in November 2002, which started in Guangdong Province, China, and spreads to 32 areas and countries, primarily in East Asia and Canada, in addition to the Middle East respiratory syndrome coronavirus (MERS-CoV) outbreak, which started in Saudi Arabia in September 2012. The clinical symptoms and epidemiological features of the three viruses are identical [3, 4]. As a result, antiviral medicines are urgently needed as vaccine programs spread worldwide. Meanwhile, the virus continues to generate novel variations that are not only more transmittable but nevertheless may also be able to evade existing vaccines. Furthermore, these vaccinations are unlikely to protect against a novel coronavirus strain. On the other hand, medication can provide far faster responses to novel viruses until specialized vaccinations can be produced. To prevent future coronavirus epidemics, new broad-spectrum antiviral treatments are required. In the current review, we assembled information on the innate nucleic acid-sensing paths predicted to be triggered by SARS-CoV-2 and the immune system evasion strategies they have established to enhance viral replication and infection. We also explore the rationale for therapeutic methods targeting these sensing pathways and their implications for COVID-19 therapy.

## 2. Coronaviruses' Pathogenicity

So far, seven different coronaviruses have been identified as being implicated in human transmittable illnesses. Four of seven coronaviruses cause common colds that infect the upper respiratory tract and lead to 35% of cold epidemic viruses. The remaining three viruses, on the other hand, “SARS-CoV, MERS-CoV, and SARS-CoV-2”, are well-known to infect the lower respiratory tract, particularly severe acute respiratory distress syndrome (ARDS) and multiple organ failure (MOF) [5]. SARS-CoV-2 causes asymptomatic and moderate illness in the majority of infected individuals; nevertheless, in severe instances, respiratory signs such as ARDS and other central nervous system symptoms are prevalent [5, 6]. Pathological investigation of COVID-19 patients' afflicted lungs revealed cellular fibro-mucinous discharges producing widespread alveolar destruction in both lungs, infiltration of inflammatory mononuclear cells, and separation of type II alveolar epithelium and hyaline membranes. This is similar to the pathology of ARDS as well as the lung pathology of SARS and MERS [7]. Low levels of type I interferons are one of the symptoms of severe COVID-19 illness [8]. In the plasma of individuals affected by COVID-19 admitted to intensive care units, significant levels of proinflammatory cytokines, notably IL-6, IL-1, IL-8, and TNF and chemokines, particularly GCSF, IP10, and MCPl, as well as poor antiviral responses, were observed. This uneven immune response fails to restrict viral replication and can result in serious systemic consequences [8, 9]. Therefore, therapies targeted at regulating immune stimulation to reduce the harmful inflammatory response or enhance an antiviral cytokine response are hopeful cure options for COVID-19 patients.

### 2.1. Hierarchical Classification of Coronaviruses

CoVs belong to the Coronaviridae family, Coronavirinae subfamily, and the Nidovirales order, which have a broad host choice from birds to mammals; nonetheless, they have not yet been found in reptiles and amphibians [10, 11]. Seven members of this family have been recognized to infect human beings till period [12]. These seven members are identified to fall within two genera. The alphacoronavirus genus comprises HCoV-229E [13] and HCoV-NL63 [14], while the betacoronavirus genus embraces HCoV-OC43 [15], HCoVHKU1 [16], SARS-CoV [17], MERS-CoV [18], and recently discovered SARS-CoV-2 [19]. The final three viruses have a tremendous genetic and structural similarity; SARS-CoV-2 has 80% and 50% homology to SARS-CoV-1 and MERS-CoV, respectively [11, 20]. This review focuses on SARS-CoV-2, which belongs to the betacoronavirus genus and subgenus sarbecovirus.

### 2.2. Structure, Genome, and Proteins of Coronaviruses

Coronaviruses are a cluster of enveloped, nonsegmented, and positive-sense single-stranded RNA viruses with a 5′-cap structure and a 3′ poly(A) tail and genomes up to 26–32 kilobases in length as one of the largest RNA viral genomes [21]. They have a diameter of 80–120 nm and are spherical. The club-like projections on the viral surface known as “spikes” are the most noticeable characteristic of coronaviruses. The CoV genome encodes nonstructural (NSP), structural, and accessory proteins.

### 2.3. Nonstructural Proteins

The open reading frames of coronaviruses (ORF) 1a and 1b potentially encode the nonstructural proteins referred to as polyproteins located at the 5′-terminal of the CoV genome, establishing the viral replicase gene that encodes 16 nonstructural proteins, which are cleaved into NSP1-16 by viral proteases mandatory for genome replication; mapped at the 50 ends, they contain approximately two-thirds of the genome [22].

### 2.4. Structural Proteins

The remaining 10 kb region preceding the 3′-end encodes several structural proteins involving surface (S), envelope (E), membrane (M), and nucleocapsid (N) proteins. The nucleocapsid demonstrates that the viral genome is surrounded by the viral envelope comprising the M and E proteins. The S glycoprotein that protrudes from the viral envelope is a class 1 viral fusion protein processed by host proteases into two functional domains, the S1 receptor binding unit and the S2 membrane fusion unit [23].

### 2.5. Accessory Proteins

**Figure 1:**
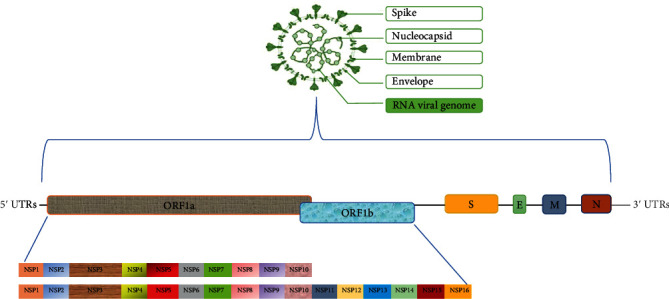
Structure, genome, and proteins of coronaviruses.

The accessory proteins ORF 3a, ORF 3b, ORF 6, ORF 7a, ORF 7b, and ORF 8 (or ORF 8a and ORF 8b in some isolates) have been recognized to be complicated in immune evasion [24]. This promotes viral pathogenesis by inhibiting type I interferons (3b and 6), modulating cellular DNA synthesis (6 and 8b), inducing apoptosis (3a, 3b, and 8a), stimulating chemokine production (3a), activating arms of the unfolded protein response, and inducing inflammatory reaction [25, 26]. Even though the accessory proteins of CoVs are not vital for viral replication and virion assembly, they lead to virulence by influencing the pathogenesis of the virus [27] (Figure 1).

## 3. Replication of SARS-CoV-2

SARS-CoV-2 affects the ACE2 receptor in humans, which is essential for cardiac activity and blood pressure regulation. The spike protein of the viral envelope's crown-like projection binds to the receptor in COVID-19 instances, and the virus's RNA genome enters the cell [28]. Because ACE2 receptors are present in almost 83% of alveolar epithelial cells, SARS-CoV-2 infection primarily influences the respiratory tract and alveolar epithelial cells. Nevertheless, these receptors are also found on the cellular surfaces of the cardiac, kidney, vascular endothelium, and gastrointestinal system [29].

The SARS-CoV-2 genome replicates in the cytosolic part of the cells. Like other positive-strand RNA viruses, this replication mechanism necessitates creating a negative-strand RNA template to amplify the positive-sense viral genome. As an intermediary in replication, this mechanism generates double-strand (ds) RNAs, which could be detected by cytosolic immune receptors and trigger a cascade of antiviral pathways [30]. These pathways activate the expression of type I/III IFNs and further innate proinflammatory cytokines, along with the oligoadenylate synthetases (OAS)-RNase L and protein kinase R (PKR) path [31]. IFNs can activate numerous downstream IFN-stimulated genes (ISGs) in the host cell, resulting in an “antiviral state” that clears viral infection [32]. Furthermore, coronaviruses reproduce within virus-stimulated cytosolic double-membrane vesicles, which shield dsRNA from detection by cytosolic receptors [33]. Additionally, the RNA of the virus, which has a 5′-cap and a 3′ poly(A) sequence, serves as messenger RNA (mRNA), which is translated by the host ribosome machinery into 16 nonstructural proteins to start infection as soon as the viral nucleic acid enters the cytoplasm [34]. The rapid replication and dissemination of SARS-CoV-2 imply a successful escape of human innate immune responses, albeit the viral proteins that cause this immune evasion remain unknown.

## 4. Function of the Innate Immune System in Viral Infection

Innate immunological interactions in the host effectively prevent viral transmission between species [11, 35]. Pattern-recognition receptors (PRRs) are found on plasma membranes, endosomal membranes, and in the cytosol. They are responsible for recognizing viral components such as viral genomic material, protein, lipids, lipoproteins, glycoproteins, structural constituents, or replication intermediates such as single and/or double-stranded RNAs, which are known as pathogen-associated molecular patterns (PAMPs). Immune cells produce many types of PRRs that directly detect viral components, including RIG-I-like receptors (RLR), toll-like receptors (TLR), NOD-like receptors (NLR), and C-type lectin-like receptors (CLmin). Furthermore, cells have likewise developed ways to indirectly identify virus infection, like nuclear or mitochondrial injury triggered by the cellular load of virus replication. Stimulators of interferon genes (STING), cyclic GMP–AMP synthase (cGAS), gamma-interferon-inducible protein 16 (IFI16) or AIM2, and DNA-dependent activator of interferon-regulatory factors (DAI) are all well-known [36, 37]. They detect dsDNA from DNA viruses.

On the other hand, they have been demonstrated to have a key function in RNA virus infection, either by straight recognizing viral signs or sensing cellular DNA released from mitochondria or nuclei due to cellular stress [38]. Substrate identification by RNA or DNA sensors triggers signalling cascades that stimulate the type I/III IFN response and the inflammatory cytokine response, two main arms of the innate immune response. The type I/III IFN pathways are directly engaged in virus spread protection and are critical for the initial cell-intrinsic antiviral interaction. The inflammatory cytokine response is important in immune cell recruitment and stimulation, which is necessary to begin an adaptive immune interaction [38].

### 4.1. IFNs Are Main Player in the Development of an Antiviral State

Type I IFNs, including IFN-*α*, IFN-*β*, IFN-*ε*, IFN-*κ*, IFN-*ω*, and type III interferon (IFN-*γ*) produced by host cells against viruses, exert a chief function in the development of an antiviral state by inducing interferon-stimulated genes (ISGs) and their proteins, which effectively suppress viral infection at equally the initial and late phases of the viral life cycle. Type I IFNs attach to their receptors, thus stimulating the JAK/STAT molecular pathway and causing ISG gene transcription [39]. Early findings on SARS-CoV-2 infection revealed that it is susceptible to type I/III IFN therapy [40–42]. Type I IFN is essential for appropriate T-cell stimulation in the initial phases of infection. Its production and response by receptor cells are highly age-dependent. Compared to older people, CD4 T-cells from youth individuals need less IFN for stimulation and survival [43]. This might clarify why old individuals are more vulnerable to SARS-CoV-2 infection [44]. In conjunction with a smaller amount of IFN described to be released in challenging subjects, this recommends that due to the similarity of SARS-CoV-1 and SARS-CoV-2 infection, they may have numerous preserved antagonistic approaches actively blocks immune activation; on the other hand, significant alterations in infection and illness could advise divergent molecular pathways.

### 4.2. Immune Evasion in Coronavirus Infection

In coronaviruses, immune evasion is accomplished by creating replication organelles, which are mostly made of twofold membrane vesicles and contain viral genomic RNA replication [45]. CoVs can avoid detection by immune receptors by modifying viral RNA to look like host mRNA [46]. Besides these passive immune evasion tactics, coronaviruses use various ways to directly target and disrupt critical immune sensors or signalling molecules [47]. The primary protease related to the virus was an effective suppressor of the cGAS-STING molecular pathways. ORF3a exhibited the distinctive capacity to inhibit STING specifically. ORF3a is found in the genetic material of the pathogenic coronaviruses SARS-CoV-2 and SARS-CoV-1 and not in the genomes of the less pathogenic betacoronaviruses HKU1 or OC43 that both contaminate humans and distinctly bind STING and suppress nuclear factor-B signalling. The chief protease of coronaviruses (3CL) of SARS-CoV-2 disrupted the formation of the STING useful complex and downstream molecular signalling by inhibiting K63-ubiquitin modification of STING [48]. This protease is in charge of the cleavage of numerous ORF1a and ORF1b polyproteins in coronaviruses and is required for virus-related replication [49]. As a result, in order to live, all viral pathogens have established effective ways of avoiding host innate immune reactions. The presence of numerous SARS-CoV-2 proteins that antagonize different human innate immune molecular pathways simultaneously highlights the relevance of host protection against SARS-CoV-2 infection [48].

## 5. Molecular Pathway of cGAS-STING against Virus Infection

**Figure 2:**
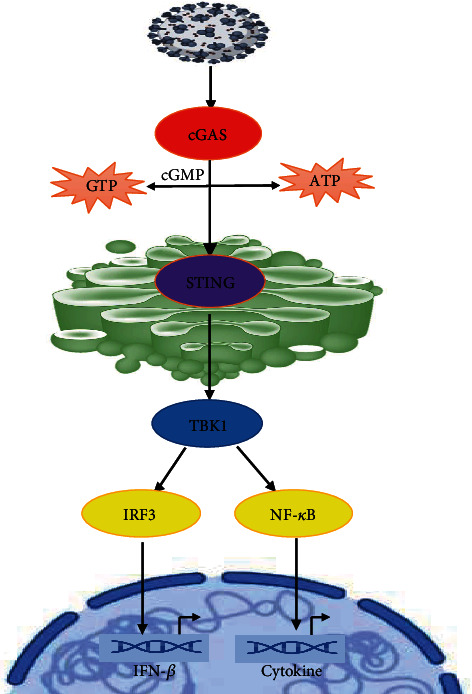
Role of STING cGAS pathway in viral infection and immunome system.

Cyclic guanosine 5-monophosphate–adenosine 5-monophosphate (cGAMP) synthase (cGAS) is an enzyme which comprises two main DNA-binding domains and a nucleotidyltransferase domain [50]. It is stimulated by attaching to double-stranded DNA (dsDNA) of DNA viruses as well as retroviruses through reverse transcription, bacteria, injured mitochondria, phagocytosed lifeless cells, and host cell chromatin through genomic instability and retrotransposons [37, 38] and converted adenosine 5-triphosphate and guanosine 5-triphosphate into the cyclic dinucleotide cGAMP [50, 51]. cGAS attaches to DNA regardless of nucleotide sequence [50], as an alternative aiming at its sugar-phosphate backbone or recognizing y-shaped constructions of ssDNAs [52, 53]. After attaching to DNA located in the cytosol, cGAS homodimerizes and produces cyclic GMP (2′-5′)-AMP (3′-5′), which attaches to and stimulates the stimulator of IFN genes (STING) (a homodimeric protein with four transmembrane domains situated at the endoplasmic reticulum (ER) or Golgi complex) and go through a conformational modification that leads to phosphorylation of the STING C-terminal, dimerization, and oligomerization [37, 38, 51]. When STING is activated, it attaches to the homodimeric TANK-binding-kinase (TBK1) and the complex oligomerizes. Afterwards, TBK1 trans-autophosphorylates stimulate and phosphorylate the STING pLxIS motif phosphorylate IFN-regulatory factor 3 (IRF3) attaches to this motif and is stimulated via a similar mechanism as TRIF to induce IFN-I and proinflammatory cytokines [38, 54] (Figure 2). After that, type I IFN attaches to its receptor, stimulating the JAK/STAT molecular pathways and promoting ISGs transcription [55]. A positive feedback loop can occur because the STING gene regards an ISG [56]. STING's serine 366 is critical for activating the TBK1–IRF3 signal axis and inducing IFN-I synthesis. In bats, this serine is found at position 358, resulting in a STING allele that produces less IFN-I [57]. As a result, bats have been found to have a greater ability to live with coronavirus [58]. DNA viruses disrupt STING's activity by cleaving or hindering its ubiquitination [59]. Although primarily a sensor for detecting DNA, STING is required for the complete regulation of RNA viruses with envelopes such as influenza and coronaviruses, independent of cGAS. STING leads to the antiviral reaction against such viruses by detecting lipid membrane fusion; therefore, STING is required for complete protection against RNA viruses [60]. Association of STING with mitochondrial antiviral molecular signalling can also engage in viral RNA sensing [60]. Through cytosolic retinoic acid-inducible gene- (RIG-I-) like sensors (RIG-I and melanoma differentiation-associated protein 5 (MDA-5)), STING additional transduces the signalling triggered by RNA-originated PAMPs [60]. In COVID-19, the STING-TBK1-IRF3 axis may be explicitly blocked by the virus's protease [47]. STING is mainly produced in endothelial cells of the lung, spleen, and epithelial cells, which are crucial for SARS-CoV-2 pathogenicity. Injured DNA spread into the cell's cytosol during the late stages of COVID-19 may stimulate the cGAS-STING molecular signalling pathway, leading to a severe cytokine storm in specific individual [61]. The lack of STING expression in specific cell types contributes to DNA virus-preferred homing. Hepatitis B virus, for example, shows a preference for human hepatocytes with untraceable amounts of STING protein [62]. Up to now, there is no applicable document that betacoronaviruses enhance STING autophagy-mediated revenue, disrupt STING trafficking, or cleave STING and/or cGAS.

On the other hand, different SARS-CoV-2 protases, NSP3, and NSP16 have been found to substantially block the downstream STING-TBK1-IRF3 molecular pathway and IFN production [47]. In a SARS-infected mouse model, fast viral transmission resulted in postponed IFN-I synthesis. It encouraged severe illness in the late stage by increasing the concentration of pathogenic monocyte-macrophages, causing lung immunopathology, vascular outflow, and inadequate T cell responses [63]. High IFN and IFN-induced chemokine concentration and strong antiviral IFN-induced gene expression were associated with initial SARS sequelae and a reduced medical outcome in SARS patients [64]. Moreover, the amount of lung injury and the concentration of cytokines in both intensive care unit and nonintensive care unit individuals in COVID-19 were more outstanding than in normal individuals [65]. Blocking abnormal activation of the cGAS-STING pathway may be viable for treating severe lung illnesses caused by SARS-CoV-1, SARS-CoV-2, or other viruses [66, 67]. The likenesses between COVID-19 and STING-associated vasculopathy with onset in infancy (SAVI) syndrome suggest that postponed STING over induction, once the self-DNA injury happens in contaminated cells, causes a harmful additional of IFN release and/or NF-*κ*B stimulation and brings about the most severe condition of the COVID-19 symptoms [68]. Furthermore, an overabundance of the STING pathway associated with ageing and metabolic diseases might explain why COVID-19 severity is linked to age, obesity, and diabetes. IFN over-reaction upon stimulation of the STING molecular pathway (overexpression in senescent cells in reaction to both abnormal cytoplasmic chromatin and/or insufficient mitochondrial DNA) is one of the processes of inflame-ageing [69]. The STING pathway also plays a role in insulin resistance and the progress of the nonalcoholic fatty liver illness. STING is currently known for its crucial role in mediating obesity-induced chronic low-grade inflammation and is activated in obese individuals [70]. Besides, the incidence and severity of myocardial infarction in individuals affected by COVID-19 and elderly persons may be clarified by delayed over-stimulation of the STING molecular pathway [71]. Recent research on SARS-CoV-2-infected K18-hACE2 transgenic mice showed that the STING pathway might have a role in this virus. Researchers discovered that endothelial cells from skin lesions from COVID-19 patients displayed signs of damage, such as loss of endothelial cell integrity, disruption of mitochondrial cristae, the release of mitochondrial DNA (mtDNA) into the cytosol, and nuclear accumulation of cleaved caspase-3, a sign of cell death. Additionally, after SARS-CoV-2 infection, endothelial cells from the lung-on-chip model showed damaged mitochondria and enrichment of mitochondrial proteins in the cytosol. The cGAS-STING pathway was activated due to the mitochondrial damage since the generation of type I IFNs may be significantly decreased by mtDNA loss. Observing dying endothelial cells, intracellular DNA foci, and broken caspase-3 pieces in IFN-producing macrophages is noteworthy. It shows a similar mechanism that activates the cGAS-STING pathway in both endothelial cells and macrophages in COVID-19 lesions [72]. Also, some other research on spike protein of SARS-CoV-2 and cGAS-STING pathway showed that S protein caused cell fusion, followed by nuclear rupture and DNA leakage into the cytoplasm. This activated the DNA sensor protein cytosolic cyclic GMP–AMP synthase (cGAS) and its downstream effector stimulator of interferon genes (STING), which boosted the expression of IFN, revealing a previously unknown mechanism of IFN response [73].

### 5.1. Cell Culture Investigations of SARS-CoV-2 Infection According to cGAS-STING

To assess if the cGAS-STING path is activated in SARS-CoV-2, Neufeldt et al. [74] evaluated alterations in the place of cGAS or STING in contaminated cells. Together, cGAS and STING were manifested to localize again to perinuclear clusters in contaminated cells, revealing stimulation. Infected cells with double staining for cGAS and dsDNA indicated that dsDNA colocalized with cGAS. They tested the influences of pharmacologically inhibiting STING in SARS-CoV-2 contaminated cells to demonstrate that cGAS-STING stimulation is complicated in the experimental increase of proinflammatory cytokines. As a result, the STING-specific inhibitor H-151 exhibited a substantially reduced TNF mRNA levels in infected cells. These findings suggest that SARS-CoV-2 contamination activates the cGAS-STING molecular pathway, resulting in NF-B-mediated production of proinflammatory cytokines and that STING inhibitors can regulate this response. Their findings suggest that cGAS-STING stimulation significantly contributes to NF-B triggering in SARS-CoV-2 contaminated cells. Because cGAS is a sensor to detect dsDNA that is not predicted to identify the genomic material of SARS-CoV-2, cellular stress or cytokine reactions caused by the contamination probably result in the secretion of nuclear or mitochondrial DNA that is detected by cGAS [75, 76]. They also demonstrated how SARS-CoV-2 contamination inhibits stimulated STING from transferring from the ER to the Golgi. It has been demonstrated that stimulation of STING at the ER is appropriate for NF-B stimulation but not for TBK1 stimulation and later IRF3 phosphorylation [77, 78]. SARS-CoV-2 infection may fragment the Golgi, impairing STING translocation to the ERGIC. SARS-CoV-2 proteins, on the other hand, might actively inhibit cGAS-STING translocation. The colocalization of STING and N protein in infected cells implies that N protein has a direct function in restricting STING translocation [74]. The alternative study by Lei et al. reported that SARS-CoV-2 contamination was unsuccessful in prompting rapid IFN-*β* production in cultured cells [79]. Surprisingly, SARS-CoV-2 causes significant IFN- production at later stages of infection [79]. These findings encouraged Zhou et al. [80] to postulate the activation of a unique signalling pathway. The cGAS-STING cytosolic DNA molecular pathway is one of the primary host innate immune molecular pathways that trigger IFN synthesis. As a result, they wondered if SARS-CoV-2 contamination activated cGAS and STING. They infected the epithelial cell line Calu-3 related to human lung and HeLa cells upregulating ACE2 (HeLa-ACE2) with SARS-CoV-2, then looked at STING phosphorylation at Ser366, which is a marker of STING activation [81]. Finally, they demonstrated that cGAS is necessary for host antiviral reactions against SARS-CoV-2 and that a STING-stimulating drug suppresses viral replication effectively [80]. In addition, Han et al. have discovered that SARS-CoV-2 ORF9b inhibits host antiviral immunity via antagonizing types I and III IFNs. SARS-CoV-2 ORF9b reduced the promoter activity of IFN-, IFN-1, and ISGs generated by various molecular elements in the RIG-I/MDA-5–MAVS, TLR3–TRIF, and cGAS-STING molecular signalling pathways, according to luciferase reporter experiments. As a result, they speculate that SARS-COV-2 may target TRIF and STING straight or signalling molecules corresponding to or downstream of the convergent points of the RIG-I/MDA5–MAVS, TLR3–TRIF, and cGAS-STING molecular signalling pathways [82].

### 5.2. Polymorphisms of the STING-Affected COVID-19

Studies have shown the role of cytokine gene polymorphisms in susceptibility to various diseases [83–85]. Several lines of evidence point to variants in the STING molecular pathway implicated in the etiology of COVID-19. The substitution of the extremely preserved and functionally essential serine residue S358 dampens STING activity. This STING mutation is linked with considerable reduced baseline IFN-b expression [57]. Surprisingly, STING variants are linked to augmented or diminished risks of age-related illnesses, and STING R293Q protects against both inflammation-ageing and obesity-related cardiovascular illness in older people [86].

## 6. Therapeutic Agents Target cGAS-STING Pathway in COVID-19

There are currently limited therapeutic opportunities for COVID-19 individuals. Remdesivir, an antiviral RNA-related polymerase inhibitor, decreases the duration of hospitalization and fatalities caused by COVID-19 [87]. Furthermore, the steroid dexamethasone has been authorized to treat severe COVID-19 cases [88]. Numerous effective vaccinations have been developed and implemented to date [89]. Despite these advancements, future endemic infections will necessitate the development of new antiviral medicines. To minimize COVID-19-related hospitalizations and fatalities, a worldwide struggle is currently proceeding to find and improve previously unidentified antiviral and anti-inflammatory medicines.

Although coronaviruses can evade host antiviral mechanisms, prestimulation of these processes can reduce coronavirus contamination and replication to some extent [90]. Early data, for example, showed that type I/III IFN pretreatment might prevent SARS-CoV-2 infection, albeit the efficacy varies among trials [42]. The document revealed that IFNs are protected quick after contamination but become pathogenic later, implying that inducing an IFN reaction early in infection is essential for preventing SARS-CoV-2 contamination, dissemination, and related pathology [47].

The cytosolic sensor of DNA upstream of STING, cGAS, is potentially a medication target for IFN-I regulation. Suramin, an antiparasitic medication, is an efficient cGAS antagonist by relocating the bound DNA from cGAS [91]. Suramin is an entrance inhibitor for various viruses, including DNA and RNA [92, 93]. Suramin is being used in clinical trials [94] and displayed control transcription of numerous inflammatory mediators, comprising type I, and type III IFNs [95]. According to a recent study, diaminobenzimidazole-based compounds are powerful, selective STING activators with improved steadiness, tissue penetrance, and efficacy above conventional cyclic dinucleotide STING agonists [96]. Although STING agonists have been found to have therapeutic implications in cancer, their antiviral potential remains unexplored. Assuming the strong type, I IFN reaction elicited by diABZI drugs. We predicted that pharmacological stimulation of STING might provide safety against SARS-CoV-2 contamination. During SARS-CoV-2 contamination, pharmacological stimulation of STING in the lung induces a fast transitory antiviral reaction via type I IFNs, NF-*κ*B–driven cytokine synthesis, and lymphocyte stimulation, causing viral replication suppression and avoidance of severe respiratory illness. The usage of diABZI-4 over alternative immunotherapies, including recombinant IFN, has numerous benefits, comprising lower price, improved permanence, room temperature storage, and the possibility of effectiveness at low doses. Although type I IFNs are required to initiate the adaptive immune reaction to SARS-CoV-2, they are insufficient to suppress SARS-CoV-2 contamination [97, 98]. Nevertheless, additional investigations on humans have shown that type I IFNs play a chief part in avoiding severe COVID-19 infection [99]. Some clinical trials supporting the usage of IFN in the initial phases of COVID-19 have revealed favorable effects [100]. STING activation induces the generation of type I IFN that mediates the stimulation of CD8+ T cell reactions.

Nonetheless, besides type I IFN reactions, STING induces NF-*κ*B and noncanonical autophagy [101, 102]. Another study used the human coronavirus OC43 animal model to investigate the influence of the STING molecular signalling pathway on coronavirus contamination. They discovered that although HCoV-OC43 contamination did not stimulate the STING molecular pathway, it successfully suppressed HCoV-OC43 contamination to a far higher level than type I interferons (IFNs). IRF3, a major STING downstream innate immune effector, was revealed to be required for anticoronavirus activation.

Furthermore, they discovered that diABZI, a human STING agonist based on amidobenzimidazole (ABZI), effectively inhibits the contamination of both HCoV-OC43 and SARS-CoV-2 [103]. They also observed that IFNs had minimal influence on HCoV-OC43 contamination; they could moderately suppress SARS-CoV-2 infection. Controversially, the human form of STING agonist diABZI might nearly entirely prevent HCoVOC43 and SARS-CoV-2 infection equally in vitro and ex vivo. As a result, compared to type I IFN pretreatment, transient activation of the STING molecular pathway has shown far more promise in preventing coronavirus contamination. diABZI may block HCoV-OC43 and SARS-CoV-2 replication by acting directly on endogenous human STING, making it an excellent treatment option for preventing infection by presently identified and future emergent coronaviruses. Their discovery that STING stimulation successfully inhibits HCoV-OC43 and SARS-CoV-2 infection imply that activating STING downstream IFN- and ISG-mediated antiviral function might be used as a new approach to combat coronavirus immune hide mechanisms. IFNs and cytokines, on the other hand, are defensive initial in infection conditions but damaging later on [32, 103]. According to reports, type I IFNs significantly influence SARS-CoV-2 infection, and their impact is dose-dependent. The immunopathology in the late stage of infection is driven by persistently elevated levels of type I IFNs. In order to effectively treat COVID-19, it is crucial to modify the type I IFN signalling system. Type I IFN responses depend heavily on the cGAS-STING pathway. According to several studies, the cGAS-STING pathway is reportedly involved in SARS-CoV-2 infection. A STING agonist called diABZI successfully prevents SARS-CoV-2 infection by evoking type I IFN responses [104].

Our findings indicate that stimulating STING and downstream innate immunity signals can be utilized to create an initial and operative host innate immune reaction for coronavirus contamination prevention.
